# Reconsidering the Cut Score of Korean National Medical Licensing Examination

**DOI:** 10.3352/jeehp.2007.4.1

**Published:** 2007-04-28

**Authors:** Duck Sun Ahn, Sowon Ahn

**Affiliations:** Department of Medical Education, College of Medicine, Korea University, Seoul, Korea.

**Keywords:** Cut Score, Standard Setting, Bookmark Method, Modified Angoff Method, Psychometrics

## Abstract

After briefly reviewing theories of standard setting we analyzed the problems of the current cut scores. Then, we reported the results of need assessment on the standard setting among medical educators and psychometricians. Analyses of the standard setting methods of developed countries were reported as well. Based on these findings, we suggested the Bookmark and the modified Angoff methods as alternative methods for setting standard. Possible problems and challenges were discussed when these methods were applied to the National Medical Licensing Examination.

## INTRODUCTION

Recently the need for reliable and valid licensing examinations for health personnel is increasing in importance in Korea. This is due to its direct relation to the quality of health personnel and subsequently the nation's health. The National Health Personnel Licensing Examination Board (NHPLEB) currently uses a cut score of 60-40% (which means 60% correct responses of overall tests and 40% correct responses of each subject) as a license requirement. These cut scores have been applied to various national examinations in Korea and are regarded as reasonable. From the perspective of psychometrics, however, it is not a valid way to set a cut score, especially for a licensing examination that is intended to discern those who acquire minimum competence from those who do not.

In this review, we first examined theories of standard setting and analyzed problems of the current cut scores. Then, we reported the results of need assessment on the standard setting among medical educators and psychometricians. Analyses of the standard setting methods of developed countries were reported as well. Based on these findings, we suggested new methods of standard setting and discussed possible problems and challenges when applied to National Medical Licensing Examination (NMLE).

## THEORIES OF SETTING CUT SCORES

A cut score indicates the minimum level of knowledge or skill that a candidate must have acquired to perform health professions. Cut scores are professionally expressed value on candidates' competency, which reflects educational philosophy and consensus among those related. This means that setting cut scores should be understood not as a mathematical technique but as a complex policy-making process.

Setting cut scores usually progresses as follows: 1) Deciding on the type of standard (absolute vs. relative standards), 2) Deciding on the method for setting standards, 3) Selecting the judges, 4) Holding the standard setting meeting, 5) Calculating the standard, 6) Checking the results [1].

There are several methods to set standards. Generally accepted methods are as follows; contrasting groups method, borderline group method, Nedelsky's method, Angoff's method, Ebel's method, and so on (for details, refer to [2]). Among these, the Angoff method had been the most popular method used for multiple-choice tests by the 1990s [3]. However, this method has a fundamental drawback, which is that it is practically impossible to estimate the probability that a minimally acceptable person would answer each item correctly. To accommodate this drawback, several modifications were suggested, one of which will be described in detail later in this paper.

## ISSUES WITH CURRENT CUT SCORES

The current 60-40% system was arbitrarily set under Japanese colonial rule, with no educational and philosophical basis. In addition, it can result in gross misclassification, such as the unfortunate failure of the competent and the fortunate pass of the non-competent depending on test difficulty.

Table 1 clearly demonstrates this phenomenon. In Table 1, the pass rate is approximately 95%. However, the pass rates of 2001 and 2003 drop to approximately 85%, lower than other years. Though not shown in Table 1, the pass rate of 1995 and 1996 was about 70%, a rate that brought mass confusion among examinees.

In principle, the difficulty of criterion-referenced test such as licensing examination is not adjusted before examination in consideration of optimal passing rate. In Korea, it is not practically possible to adjust the degree of difficulty in advance due to the drainage of test items. Instead, examiners are expected to make good questions asking basic knowledge and skill that are required to perform health professions. Since the current 60-40% criterion does not reflect this minimum level of competence, it should be changed.

## RESULTS OF NEED ASSESSMENT

Surveys were conducted to gather input from psychometricians, medical educators, and examiners. There were 38 respondents for the survey, and it was carried out from Januray 7th to 12th in 2005. We asked whether and why they thought the current cut score was valid and their suggestions for improvement. The results are in Table 2.

The followings are suggestions for improvement. First, most of the respondents believed that absolute evaluation should be retained. To do so, transformed scores can be a viable alternative. Second, examination objectives should be developed. Third, investment in faculty training for the item development is required. Fourth, scientific and systematic methodology is needed to analyze test items, to control the degree of difficulty. Fifth, item bank should hold more realistic items. Sixth, there should be flexibility in setting the cut score. For example, the Angoff method provides such flexibility. Finally, the cut score should be set under the agreement among stakeholders such as NHPLEB, medical representatives, and so on.

## STANDARD SETTING METHODS OF THE DEVELOPED COUNTRIES

We analyzed the standard setting methods of several developed countries. The results are summarized in Table 3. From the table, we can see that the basic principles of standard setting are well maintained. That is, they stick to absolute standards, and put much effort into keeping the process of standard setting rational and reasonable. These results suggest Korea should adopt the standard setting method based on scientific methods of psychometrics.

## SUGGESTIONS FOR STANDARD SETTING

We reached the agreement that Bookmark [4] and modified Angoff methods are appropriate considering the current situation in Korea. We intended to compare the two methods by applying them to real tests to increase the accuracy and validity of the results of the present study. However, we encountered a few realistic problems in carrying out such an empirical study. First, as a rule the NHPLEB does not release previous test items. Second, when releasing test items, the NHPLEB requested the research be carried out in the presence of NHPLEB personnel, which was not possible in terms of the time and resources allocated to this research. We therefore did not carry out an empirical study but instead described the process of the two methods. Both methods were recently applied to National Assessment of Educational Achievement in Korea.

### The bookmark method

In 2002, the Bookmark standard setting was applied to discern those who achieved basic competency from those who did not among 3rd year elementary school students. It was the first application of the Bookmark method in Korea. The method allows a standard setting panel to be accustomed to the characteristics of the test and provides the panel with the results of standard application. The panel consists of subject experts. For example, the panel for setting national basic performance level in reading consists of professors of Korean language education, experienced teachers and researchers, administrators, and parents. The Bookmark method proceeds as follows [5].

a) The panel received an ordered item booklet (OIB), which lists items in order of difficulty, from the easiest to the hardest. To make the OIB, item response theory (IRT) was applied and 2/3 probability of correct response was used as a scale score to represent examinee ability.
b) The panel was divided into small groups-typically groups of six to eight people. In small groups, the panel examined each item in the OIB and discussed knowledge and skills required to answer the item correctly.
c) After the group discussion, each panelist determined a cut score by placing a bookmark in the OIB based on his or her own judgment of what students with basic performance level should know and be able to do. The scale score of the marked item was the first demarcation.
d) The panel engaged in the small group discussion again to compromise differences in their opinions. The panelists then marked their choice on the OIB, which was the second demarcation.
e) Two small groups were combined into a mid-sized group to prevent one specific individual from dominating the discussion in small groups. After the group discussion, the panel was given another opportunity to change their choice and placed the bookmark on the OIB.
f) Finally, all panelists gathered in one place and discussed their choice of bookmarks. After the discussion, they were given a final opportunity to change their choice. They then placed the final bookmark on the OIB.
g) All the bookmark placements in the final round were gathered and the median was calculated to set the panel's recommended cut score.
h) Based on the cut score, the panel examined the items before the bookmark and wrote performance-level descriptors that represent a summary of the knowledge, skills, and abilities that students with the basic performance level must be able to demonstrate.

The bookmark method compensates for the drawback of the Angoff's method, which is that the panel cannot correctly estimate item difficulty. In addition, people with little psychometric knowledge can understand the meaning and implication of the cut score because the bookmark method provides performance-level descriptors. Considering these theoretical merits, combined with the practical application of the method to the diagnostic assessment of the 3rd year elementary students, the bookmark method can be a viable alternative in setting the standard of national medical licensing examination.

### Modified angoff

In the original Angoff method, there was no details of how to run an operational cut score study. Due to the lack of specificity in the original method, many modifications were suggested. The following is a general process of modified Angoff methods.

a) The choice of a panel: Generally, the panel consists of subject experts, teachers, related administrators, and so on. In case of medical licensing examination, the panel may include doctors and professors of basic science and clinic, medical administrators, and so on. The panel should be content experts and know well the characteristics of examinees. There are diverse opinions on the appropriate number of the panel, but at least 10 panelists are required, while 15~20 panelists are ideal [6-9].
b) Achievement Level Description (ALD): Conceptualizing achievement level starts from policy definition. Government agency such as the Ministry of Education & Human Resources Development or the Ministry of Health & Welfare provides a policy definition of achievement levels. Based on this policy definition, the panel discusses and describes performance of each level. This description specifies what students at each level should know and be able to do in terms of knowledge, skills, and behaviors and provides an operational definition. Exemplary items can be provided as well. To save time, the facilitator may provide preliminary ALD with the panel, so that the panel starts to set the standard with some consensus on the characteristics of a borderline group.
c) Practice: The panel practices with exemplary items. In case different types of items are mixed, they practice with each type of item. Especially for performance item, actual performance data should be provided, so that the panel can get a sense of the level of examinees.
d) The first round of estimation: The panel is divided into small groups of three or four people. The panel solves actual items on the exam. After solving the exam, they check their answers with correct ones. Then, they estimate the probability of correct answers of a borderline group of examinees for each item. After individual estimation, the results are collected and the cut score is set at the sum of medians of each item. The cut score is posted and the result of cut score application is provided, so that the panel has opportunity to check whether it is realistic and change it if necessary.
e) The second round of estimation: The panel is divided into mid-sized groups. Based on ADL and their experiences at the first round, they exchange their opinions. Then, they estimate the probability of correct answer of a borderline group of examinees for each item.
f) The third round of estimation: All the panelists gather at one place and discuss. The focus is on the items showing large deviations. After the discussion, they make third estimations. The results are collected and posted. If the third round is enough, the cut point obtained at the third round becomes the final cut score. The cut score is transformed into a scale score.
g) Description of minimum competency: If required, the panel writes what students at each level are able to do by analyzing items and students' response to the items.

The modified Angoff method is widely used in foreign counties. The method has been applied to National Assessment of Educational Achievement since 2003 by Korea Institute of Curriculum & Evaluation (for details, see [10]). Since it is based on experts' judgments and psychometric methods, the use of this method in setting the standard by the NHPLEB may enhance its expertise. In addition, the NHPLEB may be better prepared for any action taken by examinees who feel the cut score is unfair, because the method provides firm rationale behind the cut score.

### Bookmark vs. Modified Angoff Methods

The two methods are compared in terms of process, time and cost, and advantages and disadvantages. The results are shown in Table 4. Comparing all these aspects, the Bookmark method is more recommended than the modified Angoff method theoretically. However, the modified Angoff method seems more realistic in Korea considering that IRT cannot be applied to the NMLE.

## PRACTICAL ISSUES IN APPLYING THE METHODS

For the Bookmark and modified Angoff methods to be applied, there are a few practical issues to consider.

Need for committee: In a test-centered approach in setting the standards, there should be a committee for each subject. Since there are three subjects in NMLE, three committees are required. If twenty members are set for each committee, 60 members are in need, which would incur considerable costs. Insufficient budgeting, and consequent insufficient committee size, can lead to the loss of validity in setting the standard.

Need for psychometric analysis: Both methods recommended in the present paper are based on scientific analysis of psychometrics. Therefore, to apply the methods, classical item analysis and IRT should be applied in advance. For example, OIB in the Bookmark method requires pre-analysis based on IRT. The OIB costs extra time and cost, but it lessens the burden of the panel.

Need for extra cost: To apply a scientific and valid method, extra cost is inevitable.

**Fig. 1:**
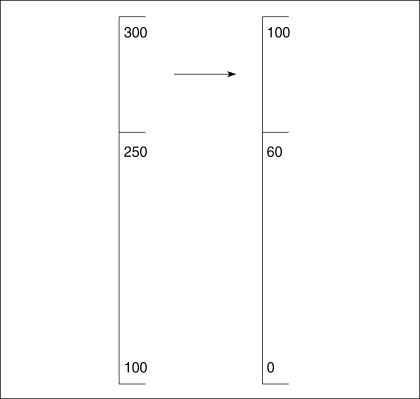
An example of the application of the current 60% cut score.

No need for amendment of medical law: A question arises as to the need for amendment of medical law with a new standard setting method. However, the current law can be maintained even with a new method. One way to do so is to transform raw scores to scale scores. In Fig. 1, the cut score of raw scores is 250, which is transformed to 60. If amendment of the law can be done without much argument, changing the current law is another way to deal with legal issues.

Test equating method: In general, the cut score is not set every year. Instead, a statistical technique is applied to make different tests comparable. This is called test equating [11, 12]. If tests are equated, the same cut score can be used continuously. However, it is not practically possible in Korea to apply test equating methods due to a drainage of test items. In this case, where the cut score is set every year, comparison of cut scores across different years would be reasonable.

## CONCLUSION

In the present paper, we attempted to suggest a valid and reasonable way to set the cut score of the NMLE. Based on the review of various theories, the need assessment, and the results of the analyses of foreign countries' systems, we suggested the Bookmark and the modified Angoff methods as viable alternatives to the current system. To apply these new methods, several issues must be resolved beforehand. First, the philosophical meaning of licensing examination itself should be reconsidered. Under the current fixed cut score, test difficulty should be adjusted in advance in order to prevent a radical change in pass rates, which violates the concept of licensing or certifying examinations.

Second, philosophical reconsideration of the standard setting is required. The current 60-40% was set without any philosophical or educational rationale. Therefore, we need to set the cut score based on the meaning and philosophy of license examination.

Third, the issue of security and copyright of test items should be resolved. Currently, the NHPLEB as a rule does not release test items. However, examinees release the items to their peers after their examination, making it possible to almost restore the original test. This is obviously infringement of copyright. In Korea, however, this kind of act is not regarded as serious and those related show no moral remorse. This common practice of plagiarism should be controlled in the near future.

We hope our research facilitates the discussion of the standard setting in Korea, and contributes to produce competent medical professionals.

## Figures and Tables

**Table 1:**
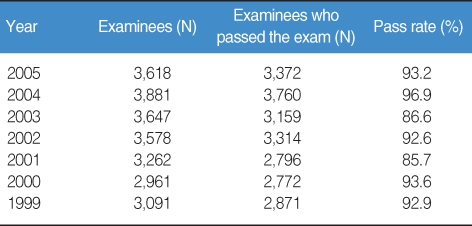
Pass rate of Korean National Medical Licensing Examination

**Table 2:**
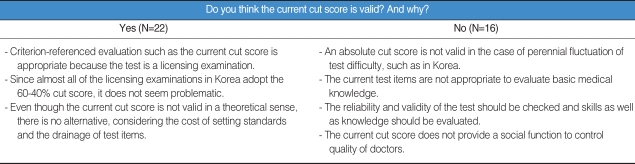
Results of need assessment (N=38)

**Table 3:**
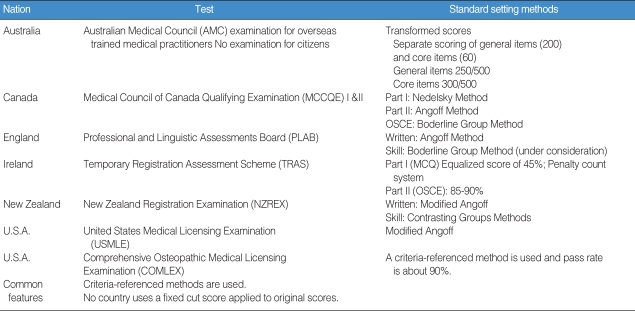
Standard setting methods of the developed countries

OSCE: Objective Structured Clinical Examination; MCQ: Multiple Choice Questions.

**Table 4:**
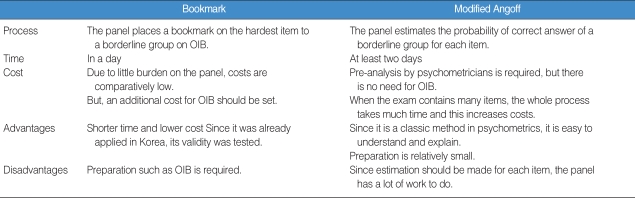
Comparison of the bookmark and modified Angoff methods

OIB: Ordered Item Booklet.
